# Actin Cytoskeleton Regulation of Epithelial Mesenchymal Transition in Metastatic Cancer Cells

**DOI:** 10.1371/journal.pone.0119954

**Published:** 2015-03-10

**Authors:** Jay Shankar, Ivan R. Nabi

**Affiliations:** Departments of Cellular and Physiological Sciences, Life Sciences Institute, University of British Columbia, Vancouver, BC, Canada; Seoul National University, REPUBLIC OF KOREA

## Abstract

Epithelial-mesenchymal transition (EMT) is associated with loss of the cell-cell adhesion molecule E-cadherin and disruption of cell-cell junctions as well as with acquisition of migratory properties including reorganization of the actin cytoskeleton and activation of the RhoA GTPase. Here we show that depolymerization of the actin cytoskeleton of various metastatic cancer cell lines with Cytochalasin D (Cyt D) reduces cell size and F-actin levels and induces E-cadherin expression at both the protein and mRNA level. Induction of E-cadherin was dose dependent and paralleled loss of the mesenchymal markers N-cadherin and vimentin. E-cadherin levels increased 2 hours after addition of Cyt D in cells showing an E-cadherin mRNA response but only after 10-12 hours in HT-1080 fibrosarcoma and MDA-MB-231 cells in which E-cadherin mRNA level were only minimally affected by Cyt D. Cyt D treatment induced the nuclear-cytoplasmic translocation of EMT-associated SNAI 1 and SMAD1/2/3 transcription factors. In non-metastatic MCF-7 breast cancer cells, that express E-cadherin and represent a cancer cell model for EMT, actin depolymerization with Cyt D induced elevated E-cadherin while actin stabilization with Jasplakinolide reduced E-cadherin levels. Elevated E-cadherin levels due to Cyt D were associated with reduced activation of Rho A. Expression of dominant-negative Rho A mutant increased and dominant-active Rho A mutant decreased E-cadherin levels and also prevented Cyt D induction of E-cadherin. Reduced Rho A activation downstream of actin remodelling therefore induces E-cadherin and reverses EMT in cancer cells. Cyt D treatment inhibited migration and, at higher concentrations, induced cytotoxicity of both HT-1080 fibrosarcoma cells and normal Hs27 fibroblasts, but only induced mesenchymal-epithelial transition in HT-1080 cancer cells. Our studies suggest that actin remodelling is an upstream regulator of EMT in metastatic cancer cells.

## Introduction

Epithelial–mesenchymal transition (EMT) is a cellular program required during normal developmental processes such as embryogenesis and tissue remodeling and also in the progression of diseases such as cancer [1]. During this process, disruption of cell-cell and cell-extracellular matrix (ECM) adhesions releases epithelial cells from the surrounding tissue. The released cells transform into mesenchymal, migratory cells with an enhanced ability to move through the meshwork of three-dimensional ECM. Localized expression of growth factors such as TGF-β and EGF induces EMT through activation of Wnt and Notch signaling pathways and downstream activation of transcription factors such as Smad, Snail, ZEB and Twist. Expression of epithelial cell–cell adhesion proteins such as E-cadherin is down regulated while mesenchymal cell–cell adhesion proteins such as N-cadherin, vimentin and the extracellular matrix proteins fibronectin and collagen, are upregulated [1,2,3].

Cortical organization of actin filaments is a hallmark of epithelial cells whereas actin stress fibres are found in mesenchymal cells. Actin cytoskeleton remodeling is mediated by the Rho GTPases and represents a basic mechanism critical to cell migration during processes such as cancer metastasis. With respect to EMT, activation of RhoA leads to ROCK-dependent actin cytoskeleton remodelling and disruption of E-cadherin based cell adhesions [4,5,6,7,8,9]. Several actin cytoskeleton–associated proteins such as α-actinin, myosin light chain, integrins, tropomyosins and moesin have been shown to be upregulated during EMT [3,7,10,11,12,13,14]. Actin cytoskeleton regulators were also identified as critical determinants of lymphoma progression in a loss-of-function RNAi screen of mouse tumor models [15]. Expression of actin regulatory proteins such as Arp2/3 and WAVE2 correlates with poor prognosis in breast and liver carcinomas supporting a role for actin cytoskeleton dynamics and organization as critical regulators of cancer progression [16]. A recent study has also implicated increased myosin IIB expression and myosin IIA heavy chain phosphorylation in enhancing mammary epithelial cell migration and invasion in TGF-β–induced EMT [17].

We showed previously that reduced expression of pseudopod-enriched proteins resulted in reduced actin cytoskeleton dynamics and cell size that were associated with a reversal of EMT in six metastatic cancer cell lines [18]. We now show that depolymerization of the actin cytoskeleton of cancer cells with cytochalasin D (Cyt D) induces nuclear-cytoplasmic translocation of EMT-associated transcription factors, increased E-cadherin expression, reduced cell shape and size and reduced activation of RhoA. In MCF-7 breast cancer cells, induction of E-cadherin by actin depolymerization requires RhoA inactivation while dominant active RhoA induces E-cadherin. This suggests that actin cytoskeleton remodeling upstream of RhoA signaling plays a role in EMT.

## Materials and Methods

### Antibodies and reagents

Mouse E-cadherin (#610182) and N-cadherin (#610920) antibodies were from BD Transduction Laboratories; anti–β-actin was from Sigma, anti-vimentin (ab11256) was from abCam. SMAD1/2/3 (Sc-7960), SNAI 1 (Sc-28199), RhoA (Sc-418), c-Myc (Sc-789) antibodies were from Santa Cruz. Alexa488–, Alexa568–, and Alexa647–conjugated secondary antibodies and rhodamine- and Alexa568-conjugated phalloidin were from Molecular Probes. Reagents for real-time PCR were from Applied Biosystems; the RNA isolation kit was from Qiagen. Cytochalasin D and Jasplakinolide were from calbiochem (Millipore). RhoA plasmids were a kind gift from Nathalie Lamarche (McGill University). RhoA beads, MLB lysis buffer and Rac1 mAb were from Millipore (Upstate).

### Cell culture, western blot and immunofluorescence microscopy

Human MDA-231, MDA-435, DU145, HT1080, U251, U87F-7, MCF-7 and Hs27 cell lines were from the American Type Culture Collection. MDA-231, MDA-435, and Du145 were maintained in complete RPMI 1640 and HT-1080, U251, U87, MCF-7 and Hs27 cells in DMEM supplemented with 10% fetal bovine serum, 100 IU/mL penicillin, 100 μg/mL streptomycin, 2 mmol/L l-glutamine, and 25 mmol/L HEPES buffer for RPM1 and sodium pyruvate for DMEM, respectively at 37°C in 5% CO_2_ incubator. For western blots, cell lysates were prepared, protein concentration was determined using Bradford reagent and equal amounts of cellular protein were loaded. For immunofluorescence, cells grown on glass coverslips were fixed with 3% paraformaldehyde and antibody-labeled as previously described [18]. Images were collected with ×60 or ×100 planapochromat objectives (numerical aperture, 1.35) of an FV1000 Olympus confocal microscope.

### Rho GTPase activity assay

RhoA pull-down was performed according to the manufacturer's protocol (Millipore) and levels of RhoA GTP and total RhoA were detected by western blotting using RhoA (Santa Cruz Biotechnology) antibody. Before treatment with Cyt D cells were transiently transfected with RhoA Plasmids using Effectene (Qiagen) according to manufacturer’s protocol.

### Cell migration and cytotoxicity assays

Approximately, 3 × 10^4^ HT1080 and HS27 cells were transferred to uncoated (migration) 8-μm cell culture inserts (BD Falcon) in medium containing 2% serum. The assembly was placed into 24-well plates containing complete medium. Cells were allowed to attach to the filter for 4–6 hrs and then treated with different concentrations of Cyt D and, as a vehicle control, 1% DMSO, for 12 hrs. After 12 hrs, non-migrating cells were removed from the top of the filter with a cotton swab and migrating cells on the bottom of the filter fixed and stained with 0.5% crystal violet, For cytotoxicity assays, approximately 7,500 cells for both HT-1080 and Hs27 were plated in 96-well plates overnight and treated with different concentrations of Cyt D or 1% DMSO for 10 hrs and then water soluble tetrazolium (WST-1 reagent) was added to the individual wells. 2 hrs after addition of the reagent, absorbance was read at 450nm. All the experiments were performed in triplicate.

### Real-time PCR

RNA was isolated from each of the cell line using the RNeasy Plus mini kit (Qiagen) and was reverse-transcribed using the High Capacity cDNA Reverse Transcription kit (Applied Biosystems). Gene expression was quantified using real-time quantitative polymerase chain reaction (qPCR) on an ABI 7500 Fast system with custom Taqman probes for each gene using Taqman Gene Expression Assays (Applied Biosystems). Relative expression was determined using a standard curve and gene expression normalized to 18S rRNA abundance.

### Statistical analysis

**Fig 1:**
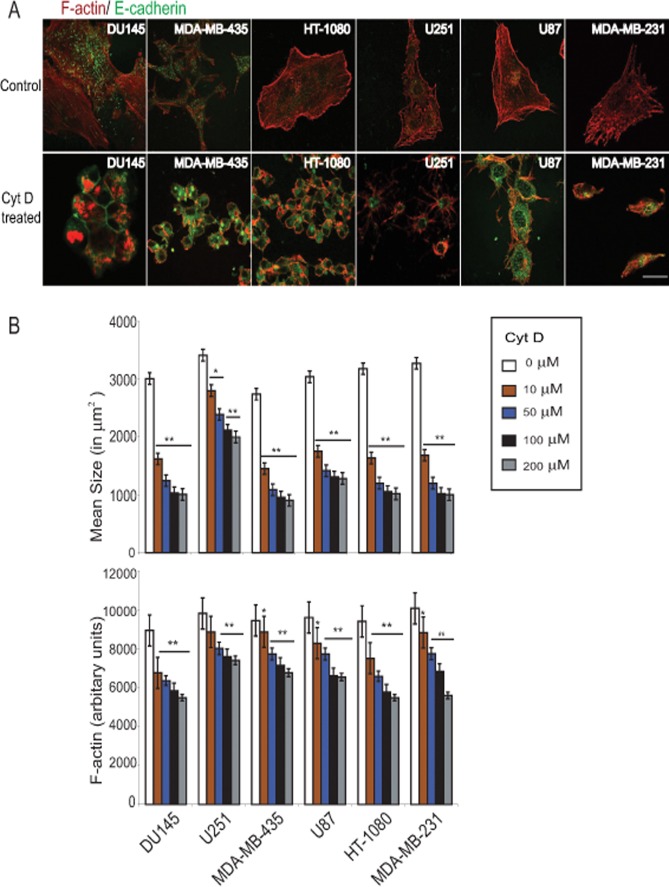
Disruption of the actin cytoskeleton with Cyt D reduces cell size and F-actin content and increases E-cadherin expression in metastatic cancer cells. (A) Du145, MDA-231, MDA-435, HT1080, U251 and U87 cells plated for 24 h were untreated (Control) or treated with 100 μM Cyt D (Cyt D treated) for 12 h, fixed and immunofluorescently labeled for E-cadherin (green) and F-actin (red). Scale: 10 μm. (B) Du145, MDA-MB-231, MDA-MB-435, HT-1080, U251 and U87 cells were treated with different concentrations of Cyt D (0, 10, 50, 100, 200 μM) and mean size and F-actin content of the cells were determined using the Morphology Explorer Bioapplication Software of a Cellomics ArrayScan VTI HCS Reader. **p>0.01 and * *p*<0.05; relative to control (untreated) cells.

**Fig 2:**
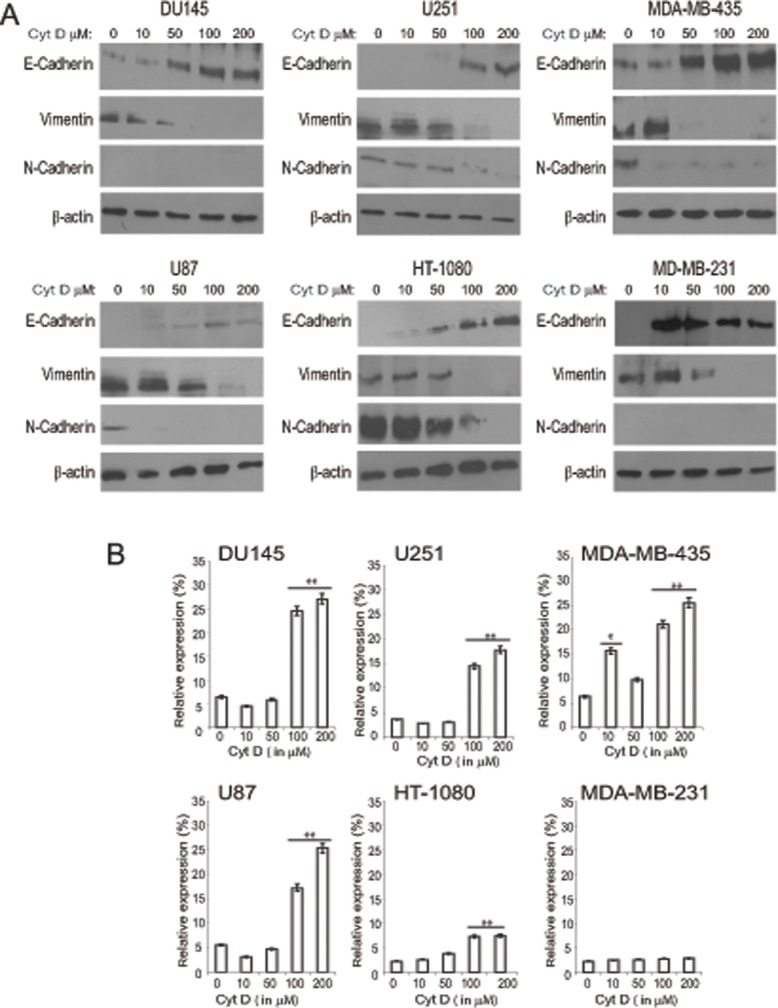
Concentration-dependent induction of E-cadherin protein and mRNA by Cyt D. (A) Du145, MDA-MB-231, MDA-MB-435, HT-1080, U251 and U87 cells were treated overnight with various concentration of Cyt D (0, 10, 50, 100, 200 μM) and cell lysates blotted for E-cadherin, N-cadherin, vimentin and β-actin. (B) Real-time PCR for E-cadherin mRNA was performed on total RNA isolated from the Du145, MDA-MB-231, MDA-MB-435, HT-1080, U251 and U87 cells treated with different concentrations of Cyt D. Cyt D treatment increased E-cadherin mRNA expression in all cells except for HT1080 and MDA-231 where there was no or minimal expression. **, *p*<0.01, * *p*<0.05; relative to control (untreated) cells.

**Fig 3:**
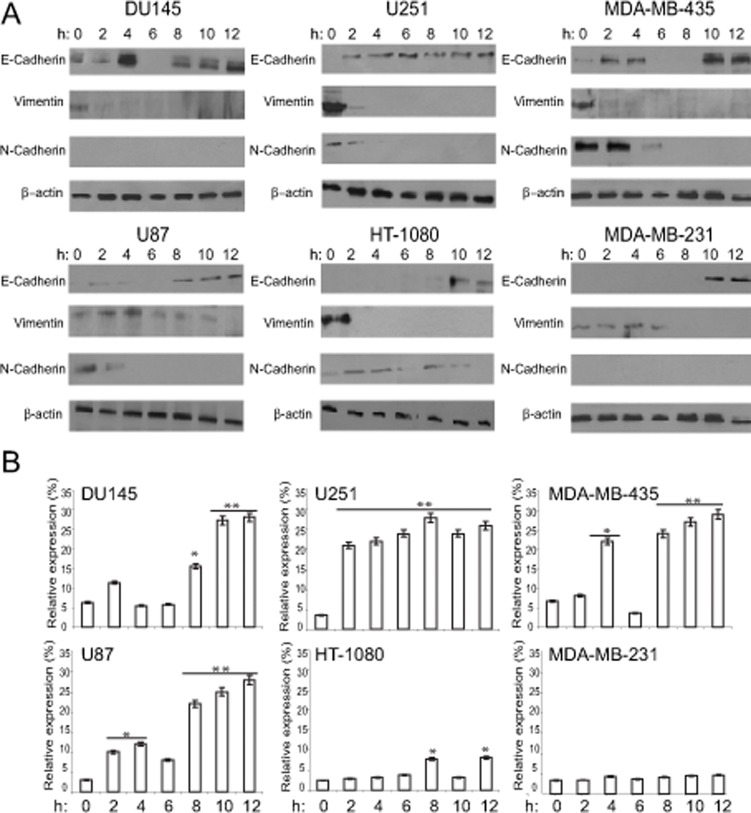
Time-dependent induction of E-cadherin protein and mRNA by Cyt D. (A) Du145, MDA-MB-231, MDA-MB-435, HT-1080, U251 and U87 cells were treated with Cyt D for the indicated times and cell lysates blotted for E-cadherin, N-cadherin, vimentin and β-actin. (B) Real-time PCR for E-cadherin mRNA was performed on total RNA isolated from the Du145, MDA-MB-231, MDA-MB-435, HT-1080, U251 and U87 cells treated at different time interval with Cyt D. Cyt D treatment increased E-cadherin mRNA expression level in all cells except for HT1080 and MDA-231 where there was no or minimal expression. **, *p*<0.01, * *p*<0.05; relative to time 0.

**Fig 4:**
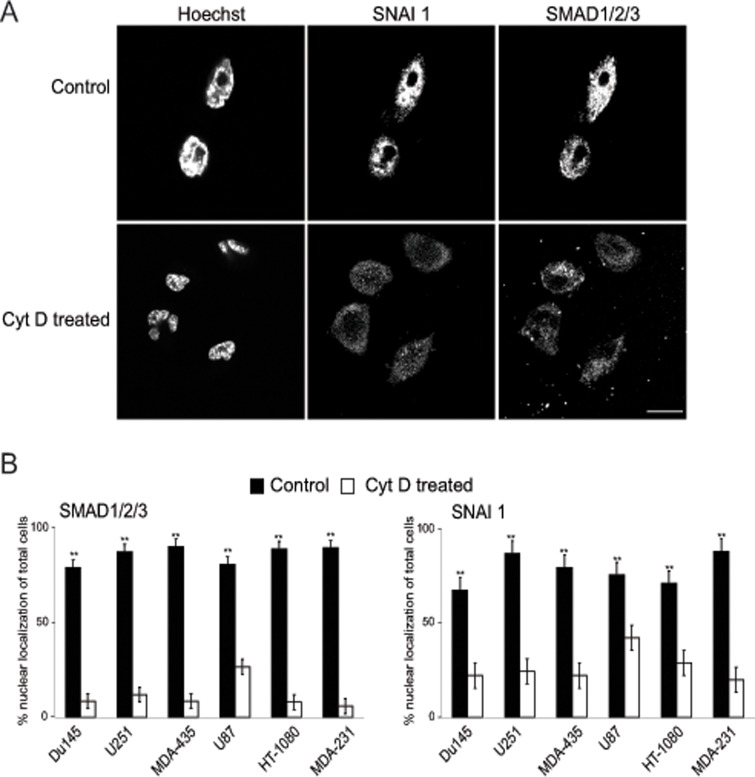
Cyt D treatment induces nuclear-cytoplasmic distribution of SNAI 1 and SMAD1/2/3. (A) Du145, MDA-MB-231, MDA-MB-435, HT-1080, U251 and U87 cells were untreated (Control) or treated with 100 μM Cyt D (Cyt D treated) for 12 h and immunofluorescently labelled for Smad1/2/3 and SNAI 1 as well as Hoechst 33342 for nuclear staining. Representative images for MDA-MB-231 cells are shown. (B) Cells were scored for nuclear distribution of Smad1/2/3 and SNAI 1 in control and Cyt D treated cells. Results are presented as percentage of total cell showing nuclear distribution for these two proteins. Scale: 10 μm; **, *p*<0.01, * *p*<0.05; relative to untreated control for each cell line.

**Fig 5:**
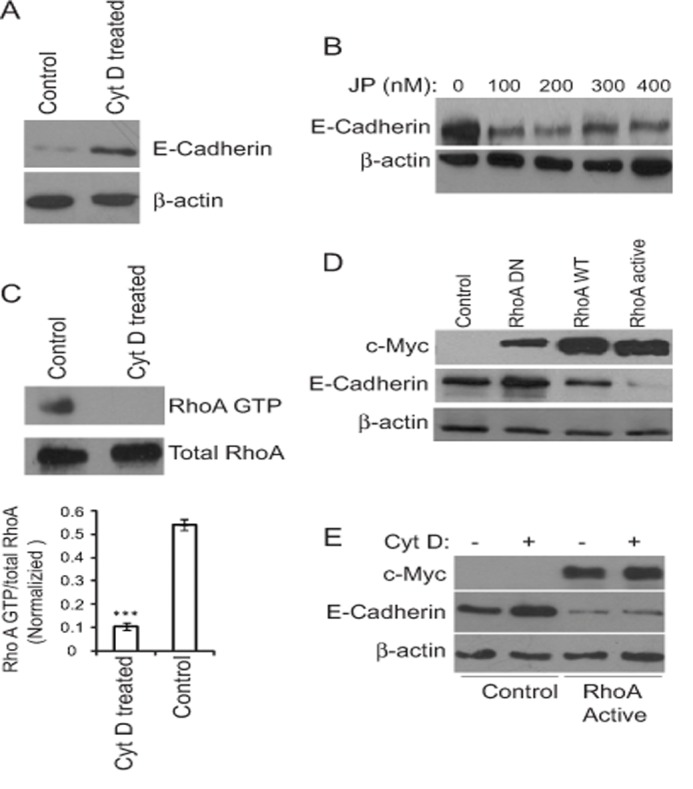
Actin polymerization regulates E-cadherin expression and RhoA GTP levels in MCF-7 cells. MCF-7 cells were treated with 100 μM Cyt D for 12 h (A) or 0–400 nM Jasplakinolide (JP) for 12 h. (B) and lysates were blotted for E-cadherin and β-actin. (C) RhoA GTP and total RhoA level were determined in MCF-7 cells treated with 100 μM Cyt D for 12 h. ***p>0.001; relative to control. (D) MCF-7 cells were transfected with dominant-negative (DN), wild type (WT) and dominant-active (DA) RhoA for 48 h after which cell lysates were probed for c-Myc, E-cadherin and β-actin. (E) Untransfected MCF-7 cells or MCF-7 cells transfected with dominant-active RhoA active were treated with 100 μM Cyt D for 12 h and cell lysates probed for c-Myc, E-cadherin and β-actin.

**Fig 6:**
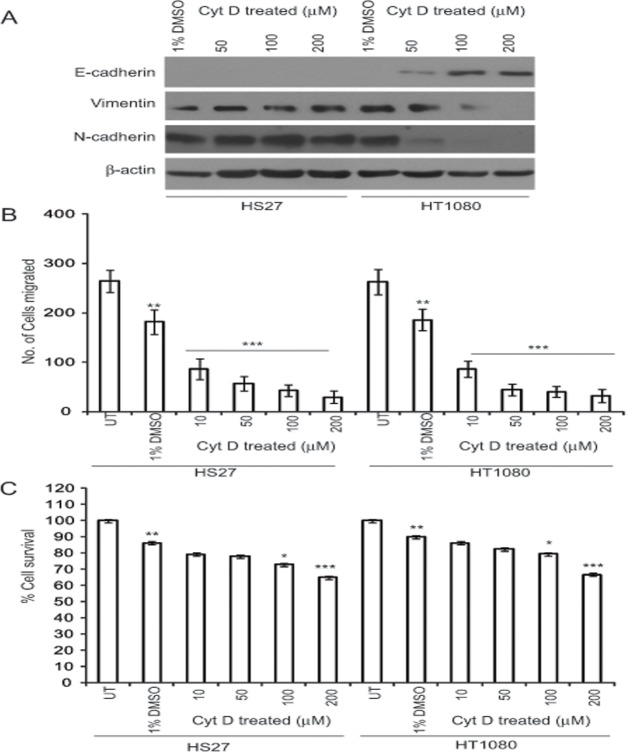
Cyt D does not induce MET in normal fibroblasts. (A) HT-1080 and Hs27 cells were treated overnight with various concentrations of Cyt D (0, 50, 100, 200 μM) and 1% DMSO as a vehicle control and cell lysates blotted for E-cadherin, N-cadherin, vimentin and β-actin. (B) For migration assays, cells plated on 8 μM cell inserts for 4–6 hrs were treated with the indicated concentrations of Cyt D for 12 hrs and the number of cell migrating through the filter counted. (C) To assess cytoxicity of Cyt D, cells were plated overnight and then treated with the indicated concentrations of Cyt D for 10 hrs. WST-1 reagent was added to each well and after two hours absorbance was read at 450 nm. *p>0.05, **p>0.01 and ***p>0.001; DMSO treated cells relative to untreated cells; Cyt D treated cells relative to DMSO treated cells.

Statistical tests were performed to determine the level of significance between control and treated groups. All the results are presented as mean ± SEM of at least three experiments. Two group comparisons (control vs treated or time points relative to time 0) were performed using the unpaired Student t test (Figs. 1, 2, 3, 4 and 5). For multiple group comparisons (Fig. 6), One-way ANOVA followed by Tukey’s multiple comparison test was performed to generate statistical data.

## Results

### The actin depolymerisation agent Cyt D induces cell shrinking and MET

Previously we reported that reduced actin cytoskeleton dynamics upon depletion of pseudopod enriched AHNAK, Septin 9, eIF4E and S100A11 in metastatic cancer cells led to increased expression of the epithelial marker E-cadherin and loss of mesenchymal markers N-cadherin and vimentin, essentially reversing epithelial-mesenchymal transition (EMT) [18]. To determine whether altering actin cytoskeleton dynamics could directly affect EMT we treated six metastatic human cancer cell lines (prostate Du145, breast MDA-MB-231 and MBA-MB-435, glioma U251 and U87 and fibrosarcoma HT-1080) with the actin depolymerising agent Cyt D. Cells exposed to Cyt D (100 μM) for 12 h showed a reduced size and stained positively for E-cadherin (Fig. 1A). Du145, MDA-MB-435, HT-1080 and U87 cells showed junctional localization of E-cadherin while U251 and MDA-231 cells presented a more intracellular E-cadherin localization (Fig. 1A). Increasing Cyt D concentrations from 10 to 200 μM resulted in progressive shrinking of the cells and reduced F-actin content, detected by fluorescent labeling with Alexa-568 phalloidin and quantitative analysis with a Cellomics ArrayScan VTI automated fluorescence imager (Fig. 1B).

Higher concentrations of Cyt D increased expression of E-cadherin and resulted in loss of mesenchymal markers vimentin and N-cadherin in all six cell lines (Fig. 2A). The most pronounced effect was observed at 100 and 200 μM Cyt D and was paralleled by increased E-cadherin mRNA, determined by qPCR, in most cell lines although to a lesser extent in HT-1080 cells and not in MDA-MB-231 cells (Fig. 2B). The time course of E-cadherin protein and mRNA induction in response to 100 μM Cyt D varied between the cell lines. For Du145, U251, MDA-MB-435 and, to a lesser extent U87, E-cadherin protein expression was induced at 2 h and associated with increased E-cadherin mRNA (Fig. 3). MDA-MB-231 and HT-1080 cells showed little or no expression of E-cadherin at the mRNA level and protein expression was only induced after 8–10 h (Fig. 3). Actin depolymerization by Cyt D therefore leads to increased E-cadherin expression at both the mRNA protein levels with induction of E-cadherin mRNA associated with more rapid induction of E-cadherin protein.

Smad1/2/3 and SNAI1 are transcription factors whose nuclear expression is associated with EMT [19] and are localized to the nucleus in the metastatic cells studied [18]. Treatment of MDA-MB-231 cells with 10 μM Cyt D resulted in the dramatic redistribution of SNAI 1 and SMAD1/2/3 to the cytoplasm (Fig. 4 A). Similar Cyt D-dependent redistribution of these two EMT-associated transcription factors from the nucleus to the cytoplasm was observed for all six metastatic cell lines studied (Fig. 4 B).

### Actin depolymerization regulates RhoA activation

Disruption of the actin cytoskeleton therefore induces an epithelial transition in metastatic cancer cells. We then tested the role of actin cytoskeleton dynamics on EMT in epithelial, non-metastatic MCF-7 breast cancer cells that express E-cadherin and have been well-characterized as a model for EMT in breast cancer cells [20]. As observed for metastatic cell lines, treatment of MCF-7 cells with Cyt D induced elevated E-cadherin levels (Fig. 5 A). In contrast, stabilization of the actin cytoskeleton with nanomolar concentrations of jasplakinolide, reduced E-cadherin levels (Fig. 5 B). Actin dynamics therefore impacts E-cadherin expression in MCF-7 cells. Activated RhoA triggers EMT [4,5,9] and induction of E-cadherin by Cyt D treatment reduced levels of active RhoA in MCF-7 cells (Fig. 5C). Consistently, transfection of MCF-7 cells with dominant-active RhoA active reduced E-cadherin expression while transfection with dominant-negative RhoA increased E-cadherin expression (Fig. 5D). Furthermore, in cells expressing dominant active RhoA, Cyt D no longer impacted E-cadherin levels (Fig. 5 E). RhoA activation status downstream of actin cytoskeleton dynamics is a key regulator of E-cadherin expression in these cancer cells.

### Cyt D does not induce MET in normal fibroblasts

To test the cancer cell specificity of Cyt D induction of E-cadherin, we treated HT-1080 fibrosarcoma cells and the normal human Hs27 fibroblast cell line with different concentrations of Cyt D (50, 100 and 200 μM) as well as with 1% DMSO, as a vehicle control, for 12 hrs. As observed before, treatment with Cyt D leads to the induction of E-cadherin in HT-1080 with concomitant loss of N-cadherin and vimentin at 50 μM Cyt D. Interestingly, treatment with Cyt D on Hs27 did not affect E-cadherin, N-cadherin or vimentin expression suggesting that Cyt D induction of MET is specific to cancer cells (Fig. 6A). Treatment with 10 μM Cyt D significantly reduced both the migration of HT1080 and Hs27 cells relative to the 1% DMSO used as a vehicle control (Fig. 6B) suggesting that cell migration is more sensitive to actin cytoskeleton disruption. Cytotoxicity assays showed that Cyt D was toxic to cells at 100 and 200 μM but not at 10 and 50 μM as compared to 1% DMSO (Fig. 6C).

## Discussion

Actin organization is critical for various cellular processes such as cell motility, cell division, organelle movement, cell signalling and the establishment and maintenance of cell junctions and cell shape [21,22]. During EMT, changes in actin organization are observed and the expression of many actin regulatory genes is upregulated [3,7,10,11,12,13,14]. We have previously reported that loss of protein components of the actin-rich pseudopodia of metastatic cancer cells alters actin cytoskeleton dynamics, reduces cell size and induces E-cadherin expression and MET [18]. Here we show that an actin depolymerising agent, Cyt D, reduces cell size and shape, inactivates RhoA and induces expression of E-cadherin, defining the actin cytoskeleton as an upstream regulator of EMT in metastatic cancer cells. Consistent with these data, we previously reported that induction of MET through loss of pseudopod-enriched proteins AHNAK, septin-9, eIF4E, and S100A11, was associated with loss of F-actin and increased stability of residual F-actin fibres [18]. Importantly, Cyt D did not induce MET in normal fibroblasts suggesting that these effects are specific for cancer cells. Whether the induction of MET by actin depolymerization is specifically related to disruption of tumor cell pseudopodia remains to be determined.

Cytoplasmic domain interaction of E-cadherin with α and β-catenin forming cell-cell adhesion complexes anchored to the actin cytoskeleton is critical to the establishment of homotypic cell-cell junctions [23]. It has been reported that treating cells with higher concentrations of Cyt D decreases cell permeability and stabilizes tight junctions [24]. It was further shown that assembly of tight junction leads to E-cadherin induction suggesting that stabilization and formation of these junctions regulate E-cadherin expression levels [25]. Earlier it was also shown that actin depolymerisation with Cyt B and latrunculin A induces surface E-cadherin expression and decreased N-cadherin and vimentin expression in rat βcells [26]. Treatment with Cyt D has also been shown to reduce vimentin expression in pancreatic cancer cells [27]. In our study, treatment of cells with Cyt D induces E-cadherin expression in all the six metastatic cell lines and also dramatically reduces cell size and shape (Figs. 1, 2). Treatment with Cyt D significantly increased E-cadherin transcript level in all but MDA-231 cells suggesting that actin cytoskeleton regulation of E-cadherin levels occurs at the mRNA and protein levels. Importantly, a more rapid induction of E-cadherin was observed in cells that also showed elevated levels of E-cadherin mRNA defining a critical role for de novo E-cadherin biosynthesis in MET. Indeed, increased levels of Zeb2, a transcriptional repressor of E-cadherin, down-regulates E-cadherin at both the mRNA and protein level and induces EMT [28].

While the most robust induction of MET was observed at higher concentrations of Cyt D (100 and 200 μM) that were associated with 10–20% cell toxicity, we consistently observed induction of MET at 50 μM Cyt D, that was not associated with significant cell toxicity and, in MDA-231 cells, at 10 μM Cyt D. Cell migration was inhibited at 10 μM Cyt D and the higher Cyt D concentrations that induced MET were also associated with increased inhibition of migration and reduced F-actin levels (Fig. 1B). We did not observe induction of MET in normal Hs27 fibroblasts at any Cyt D concentrations indicating that induction of epithelial markers by actin depolymerization is cancer cell specific. Cell migration is therefore more acutely sensitive to actin depolymerization than reversal of EMT suggesting that these two actin-based cellular responses are differentially regulated by the actin cytoskeleton.

RhoA activation can initiate EMT by promoting E-cadherin degradation [4,5,6,9,29]. Consistently, we observed that RhoA activation is associated with reduced E-cadherin levels. However, reports suggest that reduced RhoA activity is required for EMT in colon carcinoma progression [30]. In contrast to an earlier study that reported activation of RhoA by Cyt D treatment in Swiss 3T3 fibroblasts [31] we observed reduced RhoA activity in non-metastatic MCF-7 cells after Cyt D treatment that was accompanied by increased E-cadherin expression (Fig. 5). The contrasting observations might be due to the different cell lines used, fibroblasts vs epithelial MCF-7 cells, or the higher concentrations and longer incubation times used in our study. Indeed, in their study treatment was for 6 h with 0.5μg/ml (1μM), in contrast to our overnight treatment with 100 μM Cyt D, and increased activated RhoA was observed for the first 3 h only after which there was a decrease in activated RhoA levels. Reduced activity of RhoA active downstream of actin depolymerization therefore induces E-cadherin expression and MET. The fact that Cyt D does not increase E-cadherin expression levels in cells transfected with dominant active RhoA indicates that RhoA acts downstream of actin remodeling to regulate E-cadherin expression (Fig. 5) and the EMT process. Treatment of MCF-7 cells with jasplakinolide decreased E-cadherin expression suggesting that actin polymerizing and depolymerizing events have opposing effects on EMT. Interestingly, Cyt D effect seemed specific to cancer cells suggesting that small molecule regulators of actin cytoskeleton might be potentially developed as possible therapeutic agents.

Regulation of actin dynamics by pharmacologic agents can therefore affect expression of EMT markers, defining the critical role of actin dynamics in EMT and suggesting that pharmacological modulation of actin dynamics represents a valid approach to target EMT in cancer. The in vivo use of actin modulators such as cytochalasins, latrunculins, jasplakinolide and others for the treatment of cancer has been limited by their demonstrated side effects and absence of suitable delivery system for targeted delivery to tumor cells [32,33]. However recently it was shown that Cyt D encapsulated in PEG liposomes improved the solubility and bioavailability of Cyt D and induced strong anticancer effects in mouse models [34]. Identification of other compounds that can induce MET more specifically may represent alternate approaches to target the actin cytoskeleton in cancer.
