# Correction: Actin Cytoskeleton Regulation of Epithelial Mesenchymal Transition in Metastatic Cancer Cells

**DOI:** 10.1371/journal.pone.0132759

**Published:** 2015-07-13

**Authors:** Jay Shankar, Ivan R. Nabi

In the published article the β-actin blots shown for MDA-MB-231, MDA-MB-435, U251 and U87 are incorrect due to an error during preparation of the figures. The U87 β-actin blot is shown in reverse orientation; different exposures of the β-actin blot for DU145 are incorrectly shown in place of the β-actin blots for MDA-MB-231, MDA-MB-435 and U251. Here we provide a corrected Fig 3. The raw blot images are provided as a supporting information file, in which red boxes indicate blots that have been replaced in the correction, and the black boxes indicate cropped regions used for the corrected figure. This correction does not change the scientific understanding or conclusion of the paper.

**Fig 3:**
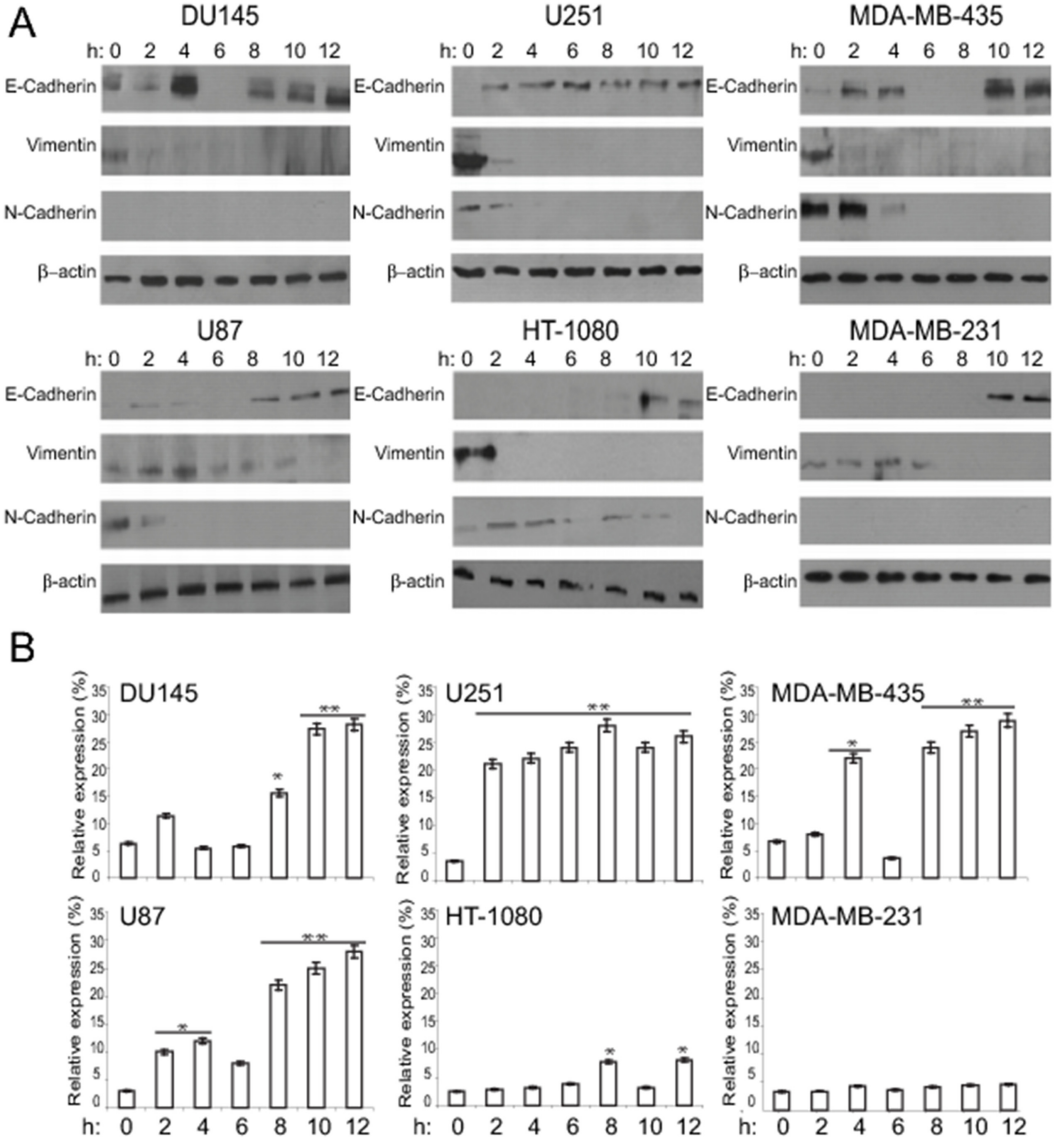
Time-dependent induction of E-cadherin protein and mRNA by Cyt D. (A) Du145, MDA-MB-231, MDA-MB-435, HT-1080, U251 and U87 cells were treated with Cyt D for the indicated times and cell lysates blotted for E-cadherin, N-cadherin, vimentin and β-actin. (B) Real-time PCR for E-cadherin mRNA was performed on total RNA isolated from the Du145, MDA-MB-231, MDA-MB-435, HT-1080, U251 and U87 cells treated at different time interval with Cyt D. Cyt D treatment increased E-cadherin mRNA expression level in all cells except for HT1080 and MDA-231 where there was no or minimal expression. **, p<0.01, * p<0.05; relative to time 0.

## Supporting Information

S1 File Raw blots. (TIF)
