# Supplementary figures and images for: Correction: Actin Cytoskeleton Regulation of Epithelial Mesenchymal Transition in Metastatic Cancer Cells
Source: PLoS One. 2015 Jul 13;10(7):e0132759. doi: 10.1371/journal.pone.0132759 (PMC4500445; doi:10.1371/journal.pone.0132759)

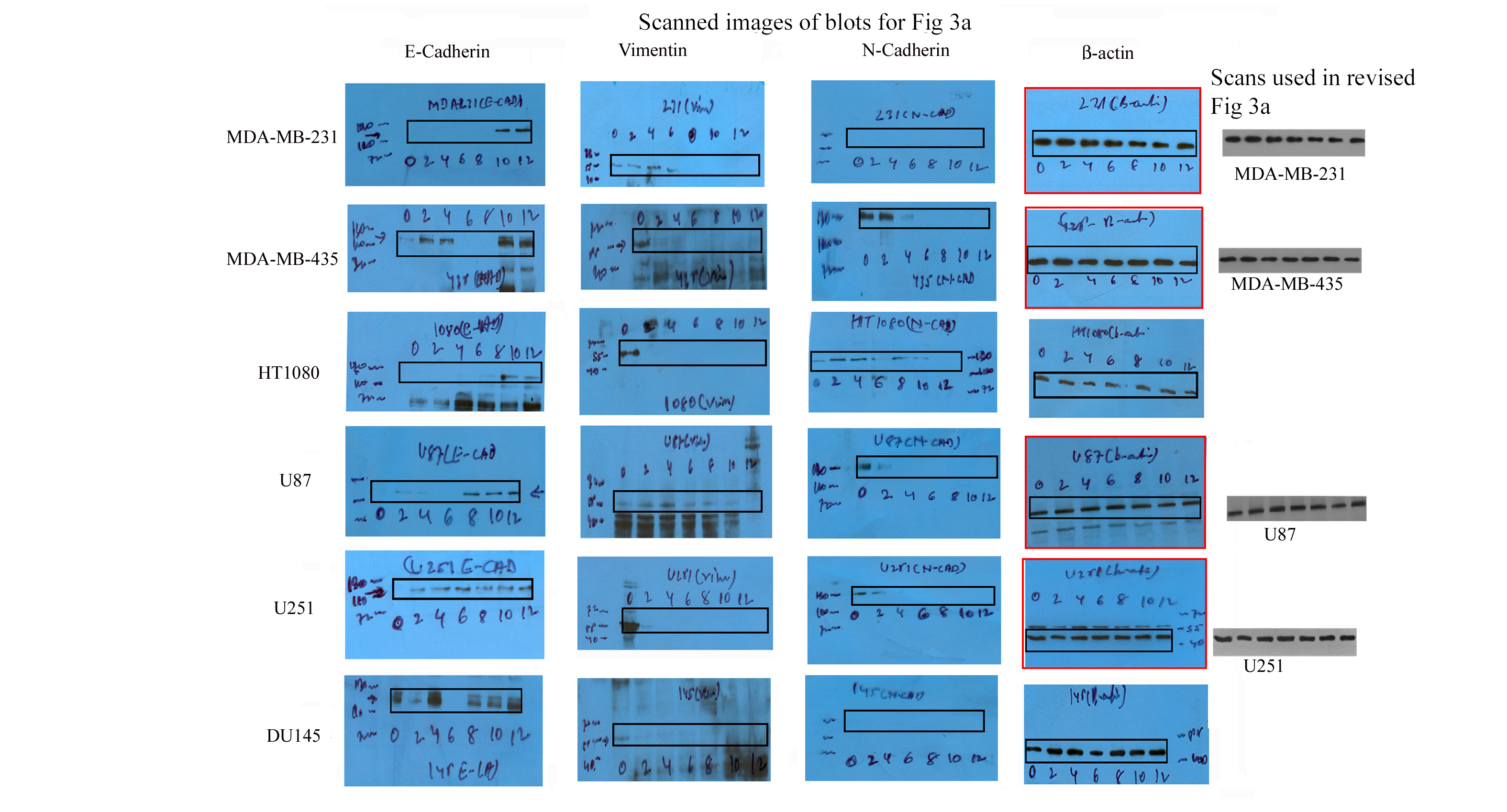

Supplement: S1 File — (TIF) [file pone.0132759.s001.tif]
